# Unstimulated Serum Thyroglobulin Levels after Thyroidectomy and Radioiodine Therapy for Intermediate-Risk Thyroid Cancer Are Not Always a Reliable Marker of Lymph Node Recurrence: Case Report and a Lesson for Clinicians

**DOI:** 10.1155/2020/8827503

**Published:** 2020-10-08

**Authors:** Luca Foppiani, Simona Sola, Manlio Cabria, Gianluca Bottoni, Arnoldo Piccardo

**Affiliations:** ^1^Internal Medicine, Galliera Hospital, Genoa, Italy; ^2^Department of Pathology, Galliera Hospital, Genoa, Italy; ^3^Nuclear Medicine, Galliera Hospital, Genoa, Italy

## Abstract

Over 50% of patients with papillary thyroid carcinoma (PTC) have cervical lymph-node metastasis on diagnosis, and up to 30% show nodal recurrence after surgery plus radioactive iodine (131I) (RAI) therapy. The combination of ultrasonography (US) and fine-needle aspiration cytology (FNAC) and the measurement of thyroglobulin (Tg) in washout fluid are cornerstones in the diagnosis of nodal metastasis. In the absence of anti-Tg antibodies, unstimulated serum thyroglobulin (Tg) levels are generally a reliable marker of recurrent disease, and 18F-FDG positron emission tomography (PET)/computed tomography (CT) plays an important role in the imaging work-up. We report the case of a 65-year-old man evaluated for a large multinodular goitre which caused compressive symptoms; the dominant nodule in the left lobe presented suspicious features on US. Thyroid function showed subclinical hypothyroidism, calcitonin was normal, serum thyroglobulin levels were low, and anti-thyroid antibodies were absent. The prevalent left nodule showed an intense uptake on 18F-FDG PET/CT but proved benign at FNAC. On the basis of the suspicious clinical and imaging features, total thyroidectomy was performed. Histology revealed a tall-cell variant of PTC with scattered expression of Tg and diffuse high expression of cytokeratin (CK) 19; RAI therapy was performed. Within 6 years of surgery, left laterocervical lymph-node recurrence was twice detected (first at levels II and III, then at levels IV and VI) by US and 18F-FDG-PET/CT and was confirmed by FNAC. Tg levels in the washout fluid proved clearly diagnostic of metastasis only in the second, larger, recurrence, whereas serum Tg levels (in the absence of anti-Tg antibodies) always remained undetectable on L-thyroxine therapy. Surgery was performed on both recurrences, and histology confirmed lymph-node metastasis of PTC. Immunohistochemical expression of Tg and CK 19 was similar to that of the primary tumour. No further relapses have occurred to date. Posttherapy (surgery and RAI) unstimulated serum Tg levels may not be a reliable marker of nodal recurrence in patients with differentiated thyroid cancer (DTC) that produces low amounts of Tg.

## 1. Introduction

DTC is the most common endocrine malignancy and generally has an excellent prognosis. Despite the hugely increased number of diagnoses over the last 20 years, mostly due to the detection of micro-PTC by the widespread use of neck US, cancer-related mortality has not risen accordingly. Cervical lymph node metastases are present in a significant percentage of patients with PTC (up to 50–60% or even 90% in the case of occult metastases) at the time of diagnosis [1].

The treatment paradigm for DTC consists of total thyroidectomy, followed by remnant ablation with RAI, and L-thyroxine therapy; the amount of TSH-suppression must be tailored according to the patient's category of risk [2]. Cornerstones of follow-up are neck US and the measurement of serum Tg levels, anti-Tg antibodies, and TSH levels.

During follow-up, US detects locoregional disease (i.e., thyroid bed or lymph nodes) in 7%–28% of PTC patients. The progression of cancer recurrence in these anatomical sites is generally slow, and the impact on clinical outcome is not well ascertained. 18F-FDG PET/CT is a reliable tool for the restaging of DTC patients who have undergone surgery and display increased (generally >10 ng/ml) Tg levels and negative neck US or whole-body scan (WBS) [3, 4]. Surgery is the reference treatment for locoregional disease [2].

We report the case of a man with a large multinodular goitre and low Tg levels, in whom the prevalent left thyroid nodule, which was negative on FNAC, proved to be a tall-cell variant of PTC on histopathology. Posttherapy (surgery and RAI) unstimulated serum Tg levels (in the absence of anti-Tg antibodies) always remained undetectable and therefore were not a reliable marker of the lymph-node recurrences that were detected over the years.

## 2. Case Report

**Figure 1:**
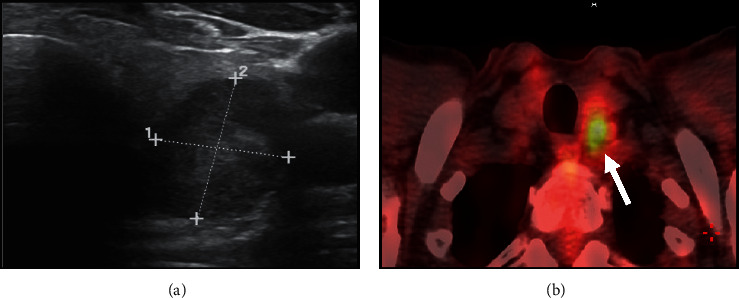
Ultrasonographic suspicious features of the large solid hypoechoic and taller-than-wide shape thyroid nodule located in the mid-inferior part of the left lobe (a), which showed significant tracer uptake at 18F-FDG PET/CT (arrow, (b)).

**Figure 2:**
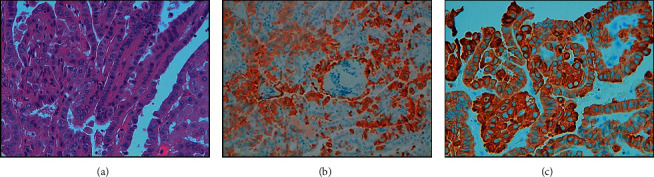
Histology after thyroidectomy showing a tall-cell variant of papillary thyroid carcinoma: papillary architecture neoplasm consisting of proliferation of elongated cells with a large eosinophilic cytoplasm, “ground glass” nuclei, and evident nuclear grooves and pseudoinclusions (haematoxylin-eosin stain, 40X, (a)). The tumour showed scattered thyroglobulin cytoplasmatic staining of different intensity (20X, (b)) and strong and diffuse CK19 cytoplasmatic staining (40X, (c)). Immunohistochemical staining was performed using specific antibodies by indirect biotin streptavidin 3, 3′-diaminobenzidine (DAB) system.

**Figure 3:**
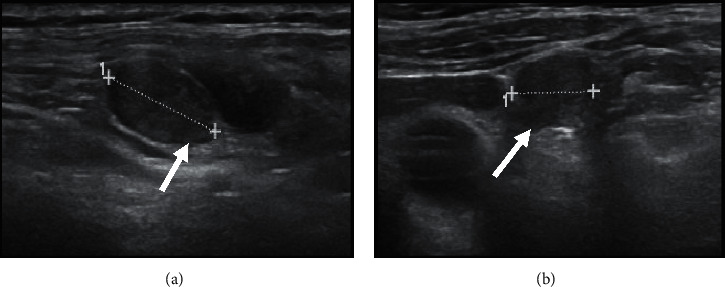
During the fourth year of follow-up after surgery and radioactive 131I therapy, ultrasonography showed small (maximum 1.5 cm) suspicious laterocervical lymph nodes with oval shape and no visible hilum (a, b, arrows).

**Figure 4:**
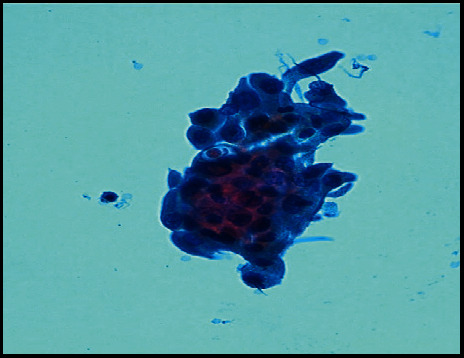
Fine needle aspiration cytology of the left laterocervical lymph-node recurrence detected during the fourth year of follow-up after surgery and radioactive 131I therapy, which showed three-dimensional papillary flaps of medium size cells with pseudoinclusions and grooves which proved compatible with metastasis of papillary thyroid carcinoma (ThinPrep method, Papanicolaou stain, 40X).

**Figure 5:**
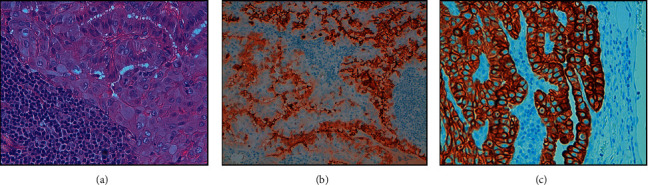
Histology of lymph node metastasis (first recurrence detected during the fourth year of follow-up after surgery and radioactive 131I therapy) of tall-cell variant of papillary thyroid carcinoma: proliferation of cells with a large eosinophilic cytoplasm, “ground glass” nuclei, and evident nuclear grooves and pseudoinclusions (left side) and residual patrimonial lymphoid tissue (right side) (haematoxylin-eosin stain, 40X, (a)). The neoplastic thyroid cells showed scattered thyroglobulin cytoplasmatic staining of different intensity (40X, (b)) and diffuse strong cytoplasmatic CK19 staining (40X, (c)). Immunohistochemical staining was performed using specific antibodies by indirect biotin streptavidin 3, 3′-diaminobenzidine (DAB) system.

**Figure 6:**
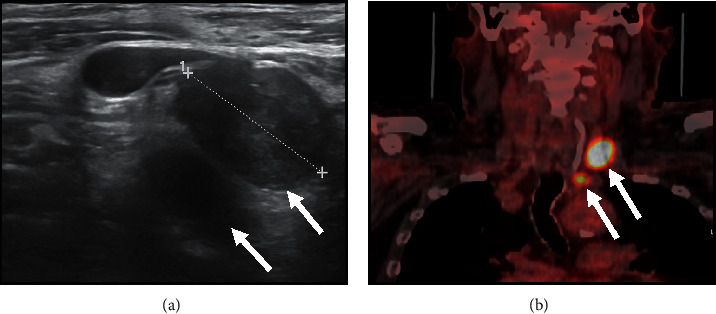
Ultrasonographic features of second nodal recurrences (6 years after surgery and radioactive iodine 131I therapy) of papillary thyroid carcinoma (two laterocervical metastatic hypoechoic lymph nodes, 2.2 cm and 1 cm, respectively) with irregular shape, no visible hilum and focal hyperechogenicity ((a), arrows). At 18F-FDG PET/CT, both lymph nodes showed significant tracer uptake (arrows) (the larger one: SUV max: 20.7; the smaller one: SUV max: 11) (b).

Eight years ago, a 65-year-old man with hypertension on therapy with candesartan 16 mg/hydrochlorothiazide 12.5 mg and benign prostatic hyperplasia treated with alfuzosin started complaining discomfort in the left part of the neck where he noticed a lump. He was evaluated by his general practitioner who palpated a large, hard left thyroid nodule and referred the patient to the thyroid out-patient clinic of our hospital. The patient reported no family history of thyroid disease or cancer. US revealed a multinodular goitre with a prevalent 4 cm hypoechoic nodule with taller-than-wide shape in the mid-lower part of left thyroid lobe which was deemed at high risk for malignancy (European Thyroid Imaging and Reporting Data System (EU-TIRADS) 5) (Figure 1(a)); no pathological lymph nodes were detected. FT4 levels were in the lower normal range: 1 ng/dl (n.v. 1.1–1.7), whereas TSH levels were slightly increased: 4.9 *μ*U/ml (n.v. 0.3–4.2), and L-thyroxine therapy (up to 75 *μ*g/day) was started. Further examinations showed normal calcitonin levels, absence of thyroid autoantibodies, and very low serum Tg levels: 1.4 ng/ml (n.v. 1.4–78). Because of the suspicious features of the left thyroid nodule, 18F-FDG PET/CT was performed, which showed intense uptake (standardized uptake value (SUV) max: 14; SUV ratio: 7.3; this latter defined as the ratio between the SUV of the nodule and that of the contralateral normal thyroid parenchyma) (Figure 1(b)). US-guided FNAC results revealed normal thyrocytes, fibrin, and histiocytes, and the nodule was classified as benign (Thyroid (Thy) 2). However, due to the clinical features, total thyroidectomy was performed. On histology, the large left thyroid nodule proved to be a tall-cell variant of PTC (pT3 pNx) which infiltrated the capsule, reached the fatty tissue, and invaded the vessels (Figure 2(a)). The tumour was deemed multifocal on the basis of the finding of another 6 mm neoplastic nodule in the right lobe. Immunohistochemistry showed scattered cytoplasmatic positivity for Tg (Figure 2(b)) and diffuse strong cytoplasmatic and membrane positivity for cytokeratin 19 (Figure 2(c)), an integral protein of the cytoskeleton of the epithelial cells which is highly expressed in PTC [5]. The thyroid tumour was classified as stage II, and the patient as being at intermediate risk of recurrence. L-thyroxine (up to 150/175 *μ*g on alternate days) was started in order to maintain TSH levels between 0.1 and 0.5 *μ*U/ml. Nearly two months after surgery, L-throxine was withdrawn until TSH levels >30 *μ*U/ml, and he was treated with ablative dose of 131I therapy (3.7 GBq). The preablative Tg level was 0.3 ng/ml, and anti-Tg antibodies were negative. Posttherapy whole-body scan (WBS) was performed 2 days after the administration of 131I and did not show any uptake. Over the first three years following surgery/RAI while on suppressive L-thyroxine therapy, Tg levels were undetectable (ultrasensitive immunoassay, analytical sensitivity: 0.04 ng/ml), and anti-Tg antibodies were absent. During the fourth year of follow-up, neck US revealed a few (diameter 1.0–1.5 cm) oval laterocervical lymph nodes, with no visible hilum and focal hyperechogenicity at levels III and IV (Figures 3(a) and 3(b)), which aroused the suspicion of recurrence; serum Tg levels on L-thyroxine therapy and anti-Tg antibodies remained undetectable. An rh-TSH stimulation test (0.9 mg intramuscularly on two consecutive days) did not result in increase in Tg levels (baseline <0.04 ng/ml, +72 h: 0.3 ng/ml). FNAC was performed on the prevalent lymph node, together with the assay of Tg levels in 1 ml of normal saline washout fluid. Washout Tg levels proved “negative” (0.24 ng/ml); by contrast, FNAC showed epithelial cells with irregular nuclei and nuclear pseudoinclusions suggestive of PTC metastasis (Figure 4). Left laterocervical lymphadenectomy (levels II, III, and IV) was performed, and 21 lymph nodes (8 of levels II and 13 of levels III and IV) were removed; on histopathology, one of the level III nodes and one of the level IV nodes showed PTC metastasis (maximum diameter 1.5 cm) (Figure 5(a)) and had scattered positivity for Tg (Figure 5(b)) and diffuse strong positivity for CK 19 (Figure 5(c)). The next year following lymphadenectomy, neck US was unremarkable, and serum Tg levels remained undetectable. Nevertheless, two years after the first recurrence, a left supraclavicular lymph node was palpated. US confirmed a 20 mm pathological (hypoechoic, with irregular shape, no visible hilum, and focal hyperechogenicity) lymph node in the left supraclavicular region (level IV) (Figure 6(a)) and a smaller one (10 mm) with the same features in the paratracheal region (level VI). 18F-FDG PET/CT showed significant tracer uptake (SUV max: 20.7) by left supraclavicular lymph node and, to a lesser extent (SUV max: 11), by left paratracheal lymph node (Figure 6(b)). FNAC of the left larger supraclavicular lymph node showed epithelial cells with enlarged and hyperchromatic nuclei that were diagnostic of metastatic PTC; this diagnosis was strengthened by the finding of increased Tg levels: 220 ng/ml in the washout fluid, in the presence of undetectable serum Tg levels. Lymphadenectomy (level IV and level VI) was performed, and histology confirmed the cytological findings. Imaging procedures six months (neck US and 18F-FDG PET/CT) and one year later (neck US) were negative, and unstimulated serum Tg was undetectable. The patient is healthy, and imaging and hormonal follow-up have been scheduled.

## 3. Discussion

More than half of patients with PTC have cervical lymphatic spread at the time of diagnosis [1, 6]. In the follow-up after surgery and RAI (when performed), US is the cornerstone technique for evaluating locoregional recurrence (thyroid bed and lymph nodes), which occurs in up to 30% of patients [7]. Two-thirds of patients show tumour recurrence within 5 years after surgery [8]. The evaluation of Tg levels in the washout fluid from FNA, the values of which correlate positively with TSH levels and serum Tg levels, improves the accuracy of the diagnosis of nodal metastasis from PTC when used in combination with FNAC [9].

The ATA [3] staging system enables the initial risk of recurrence to be restratified as a function of response to therapies (surgery and RAI if performed). The rate of recurrence may be downgraded to as low as 2% in intermediate-risk PTC patients, when the initial treatments yield an excellent response to therapy (i.e., no evidence of structural disease, unstimulated Tg < 0.2 ng/ml, or stimulated Tg < 1 ng/ml) [10]. Using these criteria, our patient, who had been categorized as being at intermediate risk of recurrence at the time of diagnosis, was subsequently downgraded to a low risk of recurrence.

US displays good sensitivity but has some limitations, such as suboptimal specificity and operator dependency [1]. In our patient, the combination of clinical examination and suspicious ultrasound features prompted us to perform 18F-FDG PET/CT in order to stratify cancer risk, which is about 35% in the case of 18F-FDG-avid nodules, while a nodule with absent/low uptake is deemed at low risk for malignancy [4].

A recent study evaluated the predictivity tests of dedicated 18F-FDG PET/CT in ninety-three patients having a thyroid nodule larger than 1 cm with US assessment of intermediate risk (EUTIRADS 4, *n* = 48) or high risk (EUTIRADS 5, *n* = 45). All patients underwent thyroidectomy, and histology was the gold standard. Overall, 18F-FDG PET/CT sensitivity and specificity were 94% and 53%, respectively. With regards to EUTIRADS 5 thyroid nodules, sensitivity, specificity, positive predictive value, and negative predictive value of 18F-FDG PET/TC were 96%, 61%, 79%, and 92%, respectively [11].

The risk of nodal recurrence is related to the presence of nodal disease at the time of initial surgery and to the number and ratio (number of positive nodes/number of nodes removed) of lymph nodes [12].

As no lymph node dissection was performed during the initial surgery, we do not know the status of nodal disease at the time of thyroidectomy.

In our patient, US detected lymph nodes suspicious for recurrences in the lateral compartment (levels III, IV, and VI) during follow-up. These locations are the most common sites of nodal spread in PTC [1]. In addition to general criteria for the detection of suspicious/tumourous lymph node, peculiar US features of lymph node metastasis from DTC include focal/diffuse hyperechogenicity, as was ascertained in our patient [13].

The two nodal recurrences, which occurred over the years, were confirmed by FNAC, which showed metastasis from PTC. US-guided FNAC of the cervical lymph nodes has high specificity; however, it yields 5–20% of inadequate results and up to 50% of false-negative results in the presence of small lymph nodes and cystic metastases [6, 14].

Serum Tg levels reflect the tumour burden or the capacity of the tumour to synthesize and secrete Tg and are used to detect recurrence of thyroid cancer after primary treatment (surgery and RAI when performed). The greatest accuracy on the basal ultrasensitive Tg assay is achieved by setting a cut-off of 0.2–0.3 ng/ml [2].

In our patient, presurgical serum Tg levels (in the absence of anti-Tg antibodies) were low and remained persistently undetectable, irrespective of two lymph node recurrences of different sizes over the years. We hypothesize that these findings were not related to reduced Tg synthesis, since no evidence of poor differentiation of the primary thyroid tumour or dedifferentiation of lymph node recurrences was reported by the pathologist. The production by the tumour of a Tg variant which was not recognized by the immunoassay cannot be excluded but seems unlikely [15].

While serum Tg is often able to indicate recurrent PTC even before the disease becomes structurally detectable on US levels, Rosario and colleagues demonstrated that serum Tg may not increase in patients with small (<1 cm) lymph node recurrence [16]. This fits well with our patient's first small nodal recurrence, but less so with the second, larger, one, in which an increase in serum Tg levels might be expected.

Overall, our findings may imply that the thyroid tumour was able to produce Tg (as shown by immunohistochemistry both in the primary tumour and in the lymph node recurrence) but had lost its functional ability to secrete it. With regards to nodal recurrences, Tg therefore remained confined within the tumoural intranodal space, and its levels in the systemic circulation subsequently remained undetectable [17]. On the rh-TSH stimulation test performed at the time of the first small recurrence, Tg showed only a minimal response vs baseline (peak <0.5 ng/ml).

In addition, patients with PTC of different histopathologic variants (included tall-cell variant) who have normal or low serum Tg (as found in our patient) before surgery may not show rising serum Tg on disease recurrence [18]. Hence, undetectable serum Tg should not be considered a reliable criterion for excluding a relatively small tumour burden in patients who have already been treated with RAI [18].

In our patient, the first laterocervical lymph-node recurrence was small (diameter 1–1.5 cm) and the very low level of Tg found in the FNA washout fluid may have been due to several factors: a procedural error, an increased clearance of Tg from plasma, loss of the immunological activity of Tg or, more likely, small amounts of Tg produced by the tumour [17]. Interference by anti-Tg antibodies in the Tg assay seems unlikely, given their absence in the serum; however, they were not measured in the FNA fluid.

A large retrospective cohort study, which evaluated Tg in the washout fluid from FNAC in 428 PTC patients by means of an ultrasensitive assay, validated 1.0  ng/ml as a cut-off value for diagnosing PTC lymph-node metastases [9], and this cut-off has been supported by a recent meta-analysis [19]. However, a universal cut-off has not yet been established, owing to the high variability among the Tg assays used [19, 20].

In this regard, Yap et al. [14] claimed that all FNA-Tg levels ≥ functional sensitivity of the assay (0.1 ng/ml in our case) were suggestive of nodal recurrence, with a high (95%) positive predictive value; if this criterion were applied, to our patient, his washout Tg levels in the first nodal recurrence would not be strictly defined as “negative.” In this setting, the marked expression of CK-19 in metastatic lymph nodes, albeit alone nonspecific for PTC [5], might strengthen the FNAC findings of recurrence and overcome the nondiagnostic result of washout Tg.

In conclusion, in the absence of anti-Tg antibodies, posttherapy unstimulated serum Tg levels may remain undetectable and hence not be a reliable marker of lymph node recurrences in DTC. This may be particularly true in DTC patients with low presurgical serum Tg levels, in whom the tumour either produces little Tg or, alternatively, has lost the capacity to secrete Tg. In these patients, US and, when appropriate, 18F-FDG PET/CT are fundamental tools for the identification of tumour recurrence.

## Data Availability

The data used to support the findings of this study are available from the corresponding author upon request.
